# Low- versus High-Chloride Content Intravenous Solutions for Perioperative Patients: A Systematic Review and Meta-Analysis

**DOI:** 10.1155/2021/3571397

**Published:** 2021-01-02

**Authors:** Xuan Song, Huairong Wang, Xinyan Liu, Xiuyan Guo, Baiqing Yu, Nana Zhang

**Affiliations:** ^1^ICU, DongE Hospital Affiliated to Shandong First Medical University, Liaocheng, China; ^2^Education Department, DongE Hospital Affiliated to Shandong First Medical University, Liaocheng, China; ^3^Emergency Department, DongE Hospital Affiliated to Shandong First Medical University, Liaocheng, China

## Abstract

**Background:**

Studies have shown complications of normal saline infusion because of its high-chloride content. Therefore, in the present study, we aimed to explore whether the use of low- versus high-chloride solutions benefited the unselected and specifically perioperative patients and was associated with different outcomes.

**Methods:**

Studies on the use of low- versus high-chloride content intravenous solutions for perioperative patients, published up to July 15, 2019, were systematically reviewed, and primary and secondary outcomes were quantitatively summarized.

**Results:**

A total of 14 eligible randomized controlled trials with 943 perioperative patients were included. Five studies reported all-cause mortality, and eight studies provided detailed data on renal replacement therapy (RRT). The pooled result suggested no statistically significant difference in the effect of low- versus high-chloride solutions on all-cause mortality (risk ratio (RR) = 1.39; 95%confidence interval (CI) = 0.23–8.26) and RRT (RR = 1.05; 95%CI = 0.63–1.76). The pooled results on acute kidney injury (AKI) and the use of allogenic blood transfusion (*P* > 0.05) were similar.

**Conclusion:**

Among specific perioperative patients, the use of low- versus high-chloride content intravenous solutions did not reduce the all-cause mortality, risk of severe AKI, or rate of RRT use. Further large randomized clinical trials are needed to confirm or refute this finding.

## 1. Introduction

Fluid therapy plays a crucial role in managing patients in the perioperative setting. However, sodium chloride (saline), one of the most commonly used and prescribed intravenous crystalloid solutions worldwide [1], has about 1.5 times chloride compared with normal plasma (95–110 mmol/L). Previous studies showed that the use of sodium chloride might contribute to hyperosmolar states [2] and hyperchloremic acidosis [3, 4]. A review published in 2008 [5] summarizing the physiological effects of hyperchloremia and acidosis indicated that hyperchloremic metabolic acidosis might impair coagulation, myocardial contractility, immune function, and renal function and even lead to mortality. Therefore, recently, the choice of intravenous fluid type for perioperative patients has gained attention [6].

Balanced or “buffered” solution has lower sodium and chloride content and a positive strong ion difference. Compared with the concentration chloride content of balanced solution, low- versus high-chloride content solutions for maintenance and fluid resuscitation were popularly used. Several randomized controlled trials (RCTs) compared the clinical outcome of patients treated with high- versus low-chloride solutions and confirmed that the balanced use of solutions could reduce the impairment of plasmatic electrolytes, acid-base equilibrium, and kidney function [7–10]. However, limited by smaller sample sizes of the included participants, several systematic reviews and meta-analyses focusing on critically ill or perioperative patients suggested that low- versus high-chloride solutions for unselected critically ill or perioperative patients demonstrated no benefits [11, 12] or a weak but significant association [13]. Moreover, Kawano-Dourado et al. searched the potential studies up to 2016 and did not provide the specific result on perioperative patients [11].

Most recently, various RCTs on this topic provided new evidence and suggested that the balanced use of crystalloids maintained the metabolic status more favorably compared with normal saline in perioperative patients, such as neurosurgical patients [14]. Therefore, the newly published literatures make it possible to finish a new systematic review and meta-analysis that is more convincing. This systematic review and meta-analysis on well-conducted and adequately powered RCTs was conducted to explore whether the use of low- versus high-chloride solutions benefited the unselected and specifically perioperative patients and was associated with different outcomes.

## 2. Material and Methods

### 2.1. Literature Search

PubMed, MEDLINE, Cochrane Library, and Embase were searched for studies published up to July15, 2019, that focused on the use of high- versus low-chloride solutions in perioperative fluid resuscitation. Target studies were selected following the Preferred Reporting Items for Systematic Reviews and Meta-Analyses (PRISMA) statement [15]. The keywords “low chloride,” high-chloride,” “balanced crystalloids,” “randomized controlled trial,” and “perioperative” were used for the literature search (Supplementary Table 1).

### 2.2. Registration

This study was registered with PROSPERO (number 42020166506).

### 2.3. Eligibility Criteria

The inclusion criteria were as follows: (1) RCTs, (2) perioperative patients as the study population, (3) use of intravenous high- versus low-chloride content solutions, (4) intravenous administration for fluid resuscitation or replacement, (5) necessary data extracted from original studies, (6) studies published in English, and (7) studies providing more detailed information if the population was reported in duplicate.

Reviews, case reports, observational studies, experimental nonrandomized studies, studies focused on animal experiments or experiments *in vitro*, and studies in languages other than English were excluded. Trials focusing on cesarean delivery or studies including participants outside the intensive care unit (ICU) were also excluded.

### 2.4. Data Extraction

All relevant studies from the databases were reviewed, and the data of included studies were extracted using a standardized form independently by two investigators (XS and XL), and the consensus was reached on all items. The extracted information included the following: study characteristics (authors, year of publication, and general information of the study population), intervention characteristics (sample size for each group and characteristics of the low- and high-chloride solutions), and outcomes (follow-up period and outcomes of each group).

### 2.5. Risk-of-Bias Assessment

The seven-category Review Manager risk-of-bias tool from RevMan (version 5.3, The Cochrane Collaboration, Oxford, UK) was used to assess the risk of bias of the studies included. The risk of bias was assessed as either high, unclear, or low according to the Cochrane Handbook for RCTs [16].

### 2.6. Quality of Study Assessment

The two reviewers evaluated the quality of evidence according to the Jadad scale, also named the Oxford quality scoring system, used to independently assess the methodological quality of a clinical trial [17]. Studies were scored according to the presence of three key methodological features: randomization, blinding, and accountability of all patients. One point or two points were added for a “yes” answer to each of the randomization and blinding, and one point was added for a “yes” to the accountability of all patients. The overall score ranged from 0 to 5. For setting a minimum standard for the results to be included in the meta-analysis, the researcher might elect to exclude all studies on the topic with a Jadad score less than 3 [18].

### 2.7. Quality of Evidence

The two reviewers evaluated the quality of evidence according to the Grading of Recommendations Assessment, Development and Evaluation (GRADE) methodology and revised Cochrane risk-of-bias tool (RoB 2.0) for quality assessment. The quality of the evidence for the included studies was assessed by GRADE Pro version 3.6 and RoB 2.0 and classified as high, moderate, low, or very low.

### 2.8. Statistical Analysis

The inverse variance method with random effects was used to summarize the dichotomous outcomes, risk ratios (RRs), and 95% confidence intervals (CIs). Stratified analyses were subsequently performed with respect to the characteristics of the study population and outcome. Heterogeneity between included studies was assessed using the *I*^2^ and *Q* tests (*P* < 0.05 was considered indicative of a statistically significant publication bias). The publication bias was assessed using the Begg rank correlation [19] and Egger weighted regression methods [20]. Forest plot generation and statistical analyses were performed using RevMan. The Begg and Egger tests were employed using Stata 15.0 (Stata Corporation, TX, USA). A *P* value of <0.05 was considered significant for all analyses.

## 3. Results

### 3.1. Study Selection

**Figure 1:**
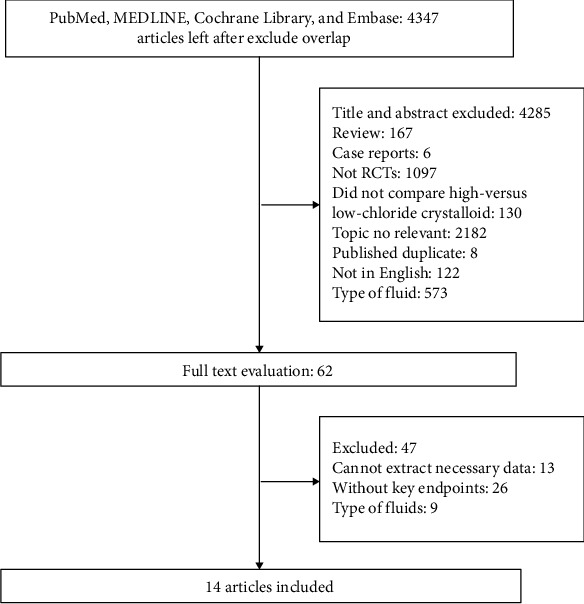
Flowchart of the study selection.

As shown in Figure 1, 4347 studies were identified by database searches with different combinations of keywords after excluding overlaps. Further, 4347 abstracts or titles were reviewed, and 4285 were excluded because they did not meet the eligibility criteria. After retrieving 47 full-length manuscripts, 9 were excluded due to the type of fluids, 26 without key endpoints, and 13 due to inability to extract necessary data. Ultimately, fourteen RCTs [7–10, 14, 21–28] with 1917 patients were finally included in this meta-analysis after retrieving 62 full-length manuscripts.

### 3.2. Study Characteristics

The characteristics of the included studies are summarized in Supplementary Table 2 and Table 1. The studies were published between 2001 and 2018, and the sample size varied from 30 to 150. The participants were from North America (two studies), Oceania (one study), Europe (seven studies), and Asia (four studies). Most studies reported the volume of fluid infused, except two studies [8, 14]. The majority of the participants were patients undergoing kidney transplantation (seven studies). Lactated Ringer was used as the low-chloride solution in seven studies, and 0.9% saline was used as the high-chloride content solution in all studies.

### 3.3. Risk of Bias and Quality of Studies

The overall risk of bias and quality of the included RCTs were acceptable (Supplementary Figure 1, Supplementary Table 3, and Supplementary Table 4). None of the included RCTs was judged to have a high risk, and most studies used a randomized and double-blinded method for including the participants.

### 3.4. Impact on Mortality

**Figure 2:**
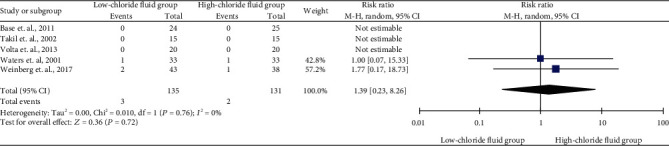
Summarized mortality and forest plot in low- and high-chloride solution groups.

Five studies reported all-cause mortality and included 5 mortalities in total (3 vs. 2 for the low- versus high-chloride solution groups). The pooled result suggested no statistically significant difference in the effect of the low- and high-chloride solutions on all-cause mortality with a summarized RR of 1.39 without heterogeneity (*I*^2^ = 0%) (95% CI, 0.23–1.21). Detailed data and forest plots are shown in Figure 2. The quality of evidence was rated as high for this outcome (Supplementary Table 5).

### 3.5. Impact on Renal Replacement Therapy

**Figure 3:**
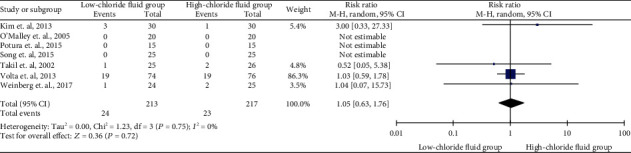
Summarized renal replacement therapy and forest plot in low- and high-chloride solution groups.

As shown in Figure 3, seven studies provided detailed data on renal replacement therapy (RRT) and included 24 events and 23 events in the low- and high-chloride solution groups, respectively. The pooled data showed no effect of low- versus high-chloride solutions on RRT and lacked heterogeneity (*I*^2^ = 0%) (RR, 1.05; 95% CI, 0.63–1.76).

### 3.6. Impact on Acute Kidney Injury and Use of Allogenic Blood Transfusion

**Figure 4:**
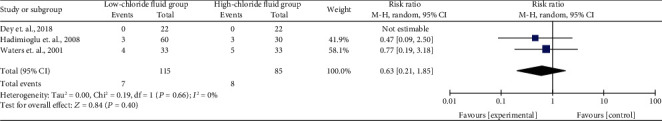
Summarized acute kidney injury and forest plot in low- and high-chloride solution groups.

**Figure 5:**
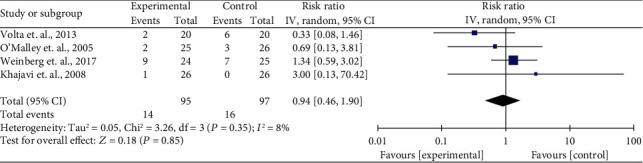
Summarized use of allogenic blood transfusion and forest plot in low- and high-chloride solution groups.

The results of the secondary primary outcomes, the acute kidney injury (AKI) and the use of allogenic blood transfusion (ABT), are shown in Figures 4 and 5. Three studies with 15 events (7 vs. 8 for the low- versus high-chloride solution groups) and four studies with 279 events (142 vs. 137 for the low- versus high-chloride solution groups) reported AKI and ABT data. Similar to all-cause mortality and RRT, no significant difference was found in the impact of the low- and high-chloride solutions on AKI and ABT with a summarized RR of 0.63 (95% CI, 0.21–1.85, *I*^2^ = 0%) and 0.94 (95% CI, 0.46–1.90, *I*^2^ = 8%), respectively. The quality of evidence was rated as high for this outcome (Supplementary Table 3).

### 3.7. Impact on Serum Potassium, pH Value, and Serum Chloride

The data on serum potassium, pH value, and serum chloride after the surgery are listed in Supplementary Table 6, Supplementary Table 7, and Supplementary Table 8, respectively. The low-chloride solution group (3.87 ± 0.25 mmol/L) had slightly lower serum potassium (*P* < 0.01, mean difference, –0.35 (95% CI, –0.71 to –0.44)) compared with the high-chloride solution group (4.63 ± 0.47 mmol/L). The low-chloride solution group (105.62 ± 0.88 mmol/L) also had lower serum chloride compared with the high-chloride solution group (112.03 ± 1.67 mmol/L), with a mean difference of –8.99 mmol/L (*P* < 0.01, 95% CI, –16.69 to –1.28).

### 3.8. Publication Bias

No potential publication bias was observed among the included trials according to the Begg rank correlation analysis and Egger weighted regression analysis (all *P* values > 0.05, Supplementary Table 9).

## 4. Discussion

Fourteen RCTs were included and summarized in the present meta-analysis addressing the use of low- versus high-chloride intravenous solutions for perioperative patients. The analysis did not find any difference in the effect of the low- and high-chloride solutions on all-cause mortality or RRT in perioperative patients. Moreover, the low- and high-chloride solutions had no effect on the secondary outcomes, AKI and ABT.

Previous meta-analyses [11] focusing on both critically ill and perioperative patients searched the potential studies from inception to October 2016 and included 15 trials. However, of the 15 trials, only nine of them provided the data on perioperative patients, and most of the included studies in the meta-analysis were small, ranging from 30 to 67 patients per study, and showed no difference in various clinical outcomes including all-cause mortality, RRT use, AKI, and ABT use between low- and high-chloride solution groups. The two most recent meta-analyses [12, 29] specifically focusing on critically ill patients indicated no difference in mortality, AKI morbidity, and RRT use between the balanced crystalloid and normal saline groups. Similar to Kawano-Dourado et al. [11], all these three studies included few RCTs with a smaller number of participants. In the current meta-analysis, we focused specifically on perioperative patients and included more RCTs. The following reasons might explain the neutral result. First, large-sample RCTs were not included in the analysis, and hence, an optimal sample size for reliable results on this topic was not achieved. Second, clinical heterogeneity should have been taken into consideration. Most included studies had variations in age and sex percentage. The comparability of the patients in the RCTs needed more attention. Third, no significant difference in primary and secondary outcomes might reflect any variability in the follow-up periods. It is also conceivable that an increased risk of AKI or other adverse outcomes may occur in long-term mortality. Therefore, inadequate sample size to detect a difference or variability in risk factors in the included patient populations might lead to an incorrect result. This meta-analysis included studies in perioperative settings; it should have also included lower-risk patients. Mortality and renal replacement therapy were the crucial evaluation indicators for the low- and high-chloride solutions in perioperative patients. In the current study, according to GRADE, the outcomes of mortality and renal replacement therapy were rated as of high quality. Therefore, the results of GRADE enhanced the persuasion of the evidence found in the current study.

Previous studies suggested that renal impairment was associated with hyperchloremic metabolic acidosis, which could be exacerbated by saline infusion [30, 31] and the acid-base changes that accompanied hyperchloremia might be related to the difference in the concentrations of strong cations (sodium and potassium), strong anions (chloride and lactic acid), and albumin. Similar to previous studies [8, 22, 23], the slightly higher serum potassium and serum chloride in the high-chloride solution group in the present study might contribute to the development of acidosis after saline administration.

Previous studies reported that fluid overload frequently occurred in perioperative patients and might be significantly associated with higher mortality and RRT [32, 33]. However, in the present study, the fluid volume varied greatly, and hence, the effects of fluid volume on perioperative patients could not be summarized. Therefore, whether exposure to a positive or negative fluid balance was detrimental remained controversial. Previous studies on this issue indicated that when a patient needed fluid resuscitation, not only fluid type but also fluid responsiveness needed to be carefully monitored [34, 35].

The limitations of the present meta-analysis should be considered while interpreting the results. First, most studies included a few patients and focused on specific populations. Due to the limited number of patients in each study, it was difficult to perform more subgroup or sensitivity analyses. Second, only a few specific patients were included in this meta-analysis, and the mean age and sex ratio of the patients varied significantly, leading to heterogeneity and reducing the stability of the results. Third, most studies did not report the severity degrees of the patients and the follow-up period also varied, again causing heterogeneity of the results. Fourth, the primary and secondary outcomes were strikingly different, and enough studies were not available to summarize more outcomes. Fifth, potential language bias might exist because the literature searches considered only studies published in English.

## 5. Conclusion

The present meta-analysis assessed the use of low- versus high-chloride content solutions on perioperative patients and did not report any difference in the effect of low- and high-chloride solutions on all-cause mortality, RRT, AKI, and ABT use. Therefore, with potential complications, the use of perioperative normal saline solution as the main infusion solution is still recommended and larger-size RCTs matched for age, sex, and severity degrees of the perioperative patients should be conducted to detect potentially important differences.

## Figures and Tables

**Table 1:** Characteristics of the included studies.

Study included	Study population	Interventions compared	Volume of fluid (liters, means ± SD) (low-/high-chloride fluid group)	Follow-up period	Outcome measures
Waters et al., 2001 [21]	Abdominal aortic, aneurysm repair	LR vs. saline	6.90 (5.70–7.90)/7.00 (5.00–8.50)^b^	In-hospital	Mortality, AKI, pH value, serum chloride
Takil et al., 2002 [22]	Spine surgery	LR vs. saline	5.10 ± 0.90/5.10 ± 1.50	12 hours	Mortality, RRT, pH value, serum chloride
O'Malley et al., 2005 [7]	Kidney transplantation	LR vs. saline	5.60 ± 1.40/6.10 ± 1.20	3 days	RRT, ABT, serum potassium, pH value, serum chloride,
Khajavi et al., 2008 [8]	Kidney transplantation	LR vs. saline	NA	Intraoperative period	RRT, ABT, serum potassium, pH value
Hadimioglu et al., 2008 [23]	Kidney transplantation	LR vs. saline	2.80 ± 0.82/2.90 ± 0.78	In-hospital	AKI, serum potassium, pH value
Base et al., 2011 [24]	Cardiac surgery	Volyt vs. Volvn^a^	2.40 ± 0.50/2.20 ± 0.50	90 days	Mortality
Volta et al., 2013	Abdominal surgery	Mixed^b^ vs. amidolite or saline	3.30 ± 0.80/2.90 ± 0.60	In-hospital	Mortality, ABT
Kim et al., 2013 [9]	Kidney transplantation	Plasma-Lyte vs. saline	3.10 ± 1.10/3.20 ± 0.90	7 days	RRT, serum chloride
Potura et al., 2015 [10]	Kidney transplantation	EIe vs. saline	2.60 (2.00–3.10)/2.50 (2.00–3.00)^b^	7 days	RRT
Song et al., 2015 [26]	Lumbar spinal surgery	Plasma-Lyte vs. saline	3.70 ± 1.60/3.20 ± 1.50	In-hospital	Mortality
Pofortmueller et al., 2017 [28]	Kidney transplantation	Mixed^c^ vs. saline	1.80 ± 0.67/1.70 ± 0.66	In-hospital	Serum chloride
Weinberg et al., 2017 [27]	Kidney transplantation	Plasma-Lyte vs. saline	1.00/1.00	48 hours	Mortality, RRT, ABT, hyperkalaemia, serum potassium, pH value, serum chloride
Pfortmueller et al., 2018 [30]	Abdominal surgery	LR vs. saline	3.10 (1.67-4.92)/3.40 (2.73-4.13)^b^	In-hospital	Hyperchloremic metabolic acidosis, catecholamine use
Dey et al., 2018 [14]	Brain tumors	Plasma-Lyte vs. saline	NA	In-hospital	AKI, serum potassium, PH value, serum chloride

LR: lactated Ringer; NA: not available; AKI: acute kidney injury; RRT: renal replacement therapy; ABT: allogenic blood transfusion. ^a^Volyt and Volvn are identical in terms of colloidal composition: both are HES 130/0.4. They differ on the amount of chloride (Volyt is a low-chloride colloid and Volvn a high-chloride solution). ^b^Range. ^c^Tetraspan and amidolite are identical in terms of colloidal composition: both are HES 130/0.42. They differ on the amount of chloride (tetraspan is a low-chloride colloid and amidolite a high-chloride solution). ^d^A chloride-reduced, acetate-buffered balanced crystalloid (Elomel Isoton ®, Fresenius Kabi Austria GmbH, Graz; osmolality 302 mOsm/kg, base excess 0 mmol/L, Na^+^ 140 mmol/L, K^+^ 5 mmol/L, Cl^–^ 108 mmol/L, Mg^++^ 1.5 mmol/L, Ca^++^ 2.5 mmol/L, acetate 45 mmol/L). ^e^Median.

## Data Availability

The datasets used and/or analyzed during the current study are available from the corresponding author on reasonable request.

## Supplementary Materials

Supplementary materials Supplementary Figure 1: risk-of-bias assessment of the included studies. Supplementary Table 1: search strategy. Supplementary Table 2: study participants' characteristics of the included studies. Supplementary Table 3: Jadad score for the included studies. Supplementary Table 4: revised Cochrane risk-of-bias tool (RoB 2.0) for quality assessment of the included studies. Supplementary Table 5: Grading of Recommendations Assessment, Development and Evaluation methodology for impacts on mortality and renal replacement therapy. Supplementary Table 6: summarized serum potassium concentration after surgery in low- and high-chloride fluid groups. Supplementary Table 7: summarized pH value after surgery in low- and high-chloride fluid groups. Supplementary Table 8: summarized serum chloride concentration after surgery in low- and high-chloride fluid groups. Supplementary Table 9: publication bias of summarized outcomes.
