# Supplementary material for: Low- versus High-Chloride Content Intravenous Solutions for Perioperative Patients: A Systematic Review and Meta-Analysis
Source: Biomed Res Int. 2021 Jan 2;2021:3571397. doi: 10.1155/2021/3571397 (PMC7801087; doi:10.1155/2021/3571397)
Supplement: Supplementary materials — Supplementary Figure 1: risk-of-bias assessment of the included studies. Supplementary Table 1: search strategy. Supplementary Table 2: study participants' characteristics of the included studies. Supplementary Table 3: Jadad score for the included studies. Supplementary Table 4: revised Cochrane risk-of-bias tool (RoB 2.0) for quality assessment of the included studies. Supplementary Table 5: Grading of Recommendations Assessment, Development and Evaluation methodology for impacts on mortality and renal replacement therapy. Supplementary Table 6: summarized serum potassium concentration after surgery in low- and high-chloride fluid groups. Supplementary Table 7: summarized pH value after surgery in low- and high-chloride fluid groups. Supplementary Table 8: summarized serum chloride concentration after surgery in low- and high-chloride fluid groups. Supplementary Table 9: publication bias of summarized outcomes. [file 3571397.f1.docx]

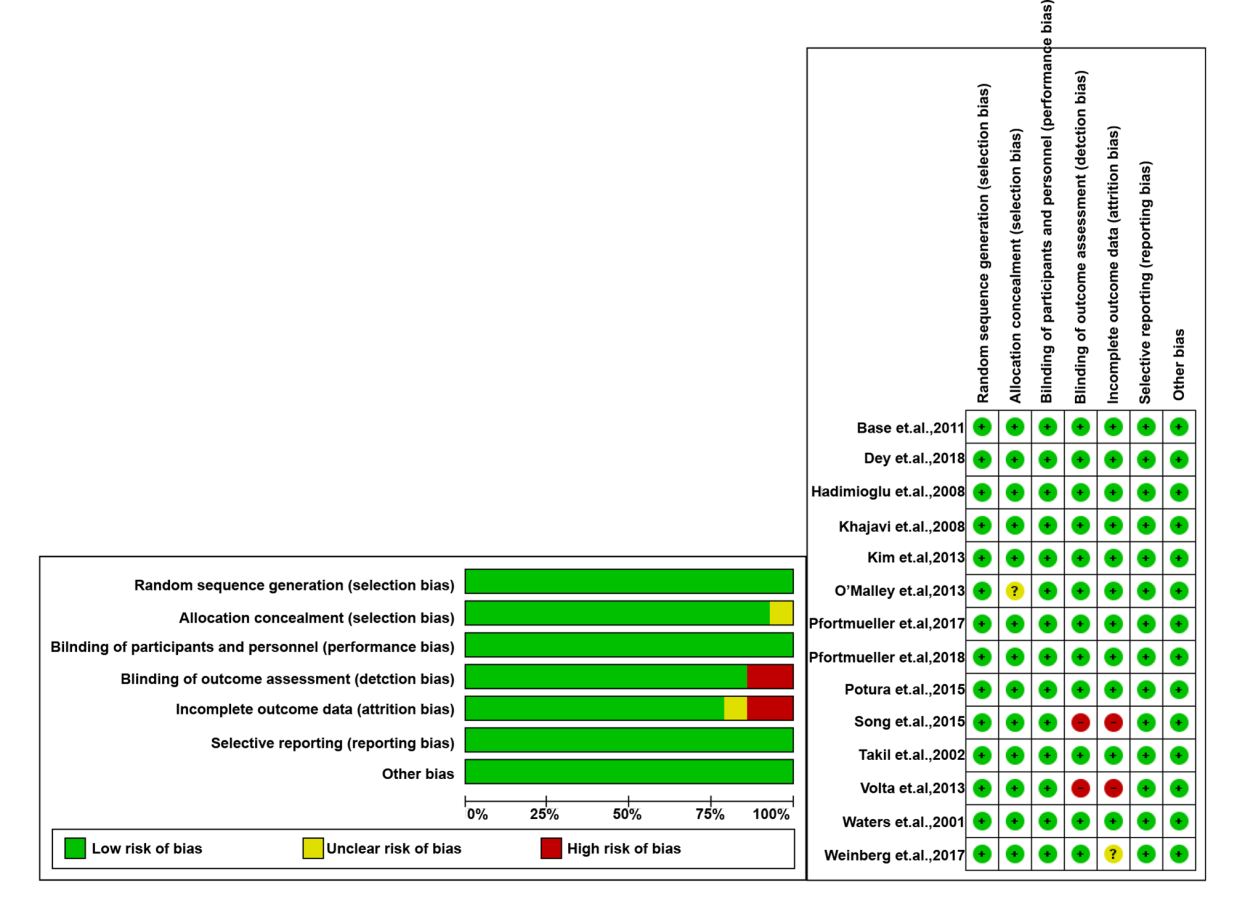


Supplementary Figure 1. Risk-of-bias assessment of included studies.

Supplementary Table 1. Search strategy

1#

" low chloride " OR "high chloride" OR "normal chloride " OR " chloride " OR " low-chloride " OR " high-chloride " OR "balanced crystalloids" OR " crystalloids" OR "crystalloid"

2#

" randomized controlled trial "[Mesh] OR " randomized controlled trial " OR " RCT" randomized controlled trials " OR " RCTs"

3#

"Perioperative Period"[Mesh] OR "Perioperative " OR "Operative Time " OR " Intraoperative " OR " Postoperative " OR " Preoperative "

Combine: 1# AND 2# AND 3#

Supplementary Table 2. Study participants’ characteristics of the included studies

| Study included | Country | Male Sex, % of subjects  (Low-/High-Chloride Fluid Group) | Age, year (means±SD)  (Low-/High-Chloride Fluid Group) |
| --- | --- | --- | --- |
| Waters et. al., 2001 | United States | NA | 69.80±8.70/ 69.90±7.80 |
| Takil et. al., 2002 | Turkey | NA | 37.00±20.00/ 45.00±19.00 |
| O’Malley et. al., 2005 | United States | 15 (60%)/ 17(65%) | 44.00±11.00/ 44.00±13.00 |
| Khajavi et. al., 2008 | Iran | NA | 37.00±13.00/ 40.00±14.00 |
| Hadimioglu et. al., 2008 | Turkey | NA | 48.30±11.50/ 44.40±12.50 |
| Base et al et. al., 2011 | Germany | 77 (77%)/ 76(76%) | 64.60 (38-83)/ 67.9 (53-83) ^a^ |
| Volta et al et. al., 2013 | Italy | 11 (55%)/ 8(36.36%) | 68.00±10.00/ 69.00±15.00 |
| Kim et al et. al., 2013 | Korea | 17 (57%)/ 21(70%) | 44.00±12.00/ 46.00±12.00 |
| Potura et. al., 2015 | Austria | 47 (64%)/ 48(63%) | 54.00±13.00/ 56.00±13.00 |
| Song et. al., 2015 | Korea | 8 (32%)/ 8(36%) | 60.00±12.00/ 63.00±10.00 |
| Pofortmueller et. al., 2017 | Austria | 47 (64%)/ 48(63%) | 54.00 ± 13.00/ 56.00 ± 13.00 |
| Weinberg et. al., 2017 | Australia | 15 (63%)/ 18(72%) | 53.00 (23–73)/ 49.00 (26–67) ^a^ |
| Pfortmueller et. al., 2018 | Austria | 11 (37%)/ 13 (43%) | 63.00/ 58.00 ^b^ |
| Dey et. al., 2018 | India | 9 (41%)/ 11 (50%) | 38.50±11.43/ 45.86±14.22 |

Abbreviations: SD, standard deviation; NA, not available.

^a^, range of age.

^b^, median of age.

Supplementary Table 3. Jadad score for included studies

| Study included | Randomization ^a^ | Blinding ^b^ | An account of all patients ^c^ | Overall |
| --- | --- | --- | --- | --- |
| Base et.al., 2011 | 2 | 2 | 1 | 5 |
| Dey et.al., 2018 | 2 | 2 | 1 | 5 |
| Khajavi et.al., 2008 | 2 | 2 | 1 | 5 |
| Kim et.al., 2013 | 2 | 2 | 1 | 5 |
| O’Malley et.al., 2005 | 2 | 2 | 1 | 5 |
| Potura et.al., 2015 | 2 | 0 | 1 | 3 |
| Song et.al., 2015 | 2 | 2 | 1 | 5 |
| Takil et.al., 2002 | 2 | 0 | 1 | 3 |
| Volta et.al., 2013 | 2 | 2 | 1 | 5 |
| Waters et.al., 2001 | 2 | 2 | 1 | 5 |
| Pfortmueller et.al., 2018 | 2 | 2 | 1 | 5 |
| Pfortmueller et.al., 2017 | 2 | 0 | 1 | 3 |
| Hadimioglu et.al., 2008 | 2 | 2 | 1 | 5 |
| Weinberg et.al., 2017 | 2 | 2 | 1 | 5 |

^a^, 1 point if randomization is mentioned, 1 additional point if the method of randomization is appropriate. Deduct 1 point if the method of randomization is inappropriate (minimum 0).

^b^, 1 point if blinding is mentioned, 1 additional point if the method of blinding is appropriate. Deduct 1 point if the method of blinding is inappropriate (minimum 0).

^c^, the fate of all patients in the trial is known. If there are no data, the reason is stated.

Supplementary Table 4. Revised Cochrane risk-of-bias tool (RoB 2.0) for quality assessment of included studies.

|  | Risk of bias arising from the randomization process | Risk of bias due to deviations from the intended interventions | Risk of bias due to missing outcome data | Risk of bias in measurement of the outcome | Risk of bias in selection of the reported result | Overall risk ^a^ |
| --- | --- | --- | --- | --- | --- | --- |
| Waters et. al., 2001 | Low | Low | Low | Low | Low | Low |
| Takil et. al., 2002 | Low | Low | Low | Low | Low | Low |
| O’Malley et. al., 2005 | Low | Some concern | Low | Low | Low | Moderate |
| Khajavi et. al., 2008 | Low | Some concern | Low | Low | Low | Moderate |
| Hadimioglu et. al., 2008 | Low | Low | Low | Low | Low | Low |
| Base et al et. al., 2011 | Low | Low | Low | Low | Low | Low |
| Volta et al et. al., 2013 | Low | Low | High | High | Low | High |
| Kim et al et. al., 2013 | Low | Low | Low | Low | Low | Low |
| Potura et. al., 2015 | Low | Low | Low | Low | Low | Low |
| Song et. al., 2015 | Low | Low | High | High | Low | High |
| Semler et. al., 2017 | Low | Low | Low | Low | Low | Low |
| Pofortmueller et. al., 2017 | Low | Low | Low | Low | Low | Low |
| Weinberg et. al., 2017 | Low | Low | Some concern | Low | Low | Moderate |
| Pfortmueller et. al., 2018 | Low | Low | Low | Low | Low | Low |
| Dey et. al., 2018 | Low | Low | Low | Low | Low | Low |

^a^, Low risk of bias: the study is judged to be at low risk of bias for all domains for this result; some concerns, the study is judged to raise some concerns in at least one domain for this result, but not to be at high risk of bias for any domain; high risk of bias: the study is judged to be at high risk of bias in at least one domain for this result. Or the study is judged to have some concerns for multiple domains in a way that substantially lowers confidence in the result.

Supplementary Table 5. Grading of Recommendations Assessment, Development and Evaluation methodology for impacts on mortality and renal replacement therapy

| Interventions for [condition] in [Population] | | | | | | |  |
| --- | --- | --- | --- | --- | --- | --- | --- |
| Outcome | Intervention and Comparison Intervention | Illustrative comparative risks* (95%Cl) |  | Relative effect (95%Cl) | No of Participants (studies) | Quality of the evidence (GRADE) | Comments |
|  |  | Assume risk with comparator | Corresponding risk  with Intervention |  |  |  |  |
| Impact on mortality | | | | | | | |
|  | Low-chloride content intravenous solution/High-chloride content intravenous solutions | Study population | | RR 1.39  (0.23 to 8.26) | 266  (5 studies) | ⊕⊕⊕⊕  High |  |
|  |  | 145 per 1000 | 134 per 1000 (103 to 176) |  |  |  |  |
|  |  | Moderate |  |  |  |  |  |
| Impact on renal replacement therapy | | | | | | | |
|  | Low-chloride content intravenous solution/High-chloride content intravenous solutions | Study population | | RR 0.76  (0.51 to 1.15) | 1344  (7 studies) | ⊕⊕⊕⊕  High |  |
|  |  | 70 per 1000 | 56 per 1000  (36 to 86) |  |  |  |  |
|  |  | Moderate |  |  |  |  |  |

Supplementary Table 6. Summarized serum potassium concentration after surgery in Low- and high Chloride Fluid Groups

| Study included | Low-Chloride Fluid Group | | High-Chloride Fluid Group | | Mean difference |
| --- | --- | --- | --- | --- | --- |
|  | Mean±SD ( mmol/L) | total | Mean±SD ( mmol/L) | total |  |
| O’Malley et.al., 2005 | 3.80±0.40 | 25 | 3.90 ±0.40 | 26 | -0.10 (-0.32, 0.12) |
| Weinberg et.al., 2017 | 5.40±0.90 | 24 | 6.10±0.80 | 25 | -0.70 (-1.1, -0.22) |
| Dey et.al., 2018 | 3.29±0.25 | 22 | 3.28±0.24 | 22 | 0.01 (-0.13, 0.15) |
| Khajavi et.al., 2008 | 4.03 ± 0.80 | 26 | 4.88 ± 0.70 | 26 | -0.85 (-1.26, -0.44) |
| Total/ Summarized concentration | 3.87±0.25 | 617 | 4.63±0.47 | 553 | -0.35 (-0.71, -0.44) |
| I ^2^ | 99.07 | / | 99.01 | / | / |
| Begg | 0.55 | / | 0.36 | / | / |
| Egger | 0.62 | / | 0.32 | / | / |

Abbreviations: SD, standard deviation.

Supplementary Table 7. Summarized PH value after surgery in Low- and high Chloride Fluid Groups

| Study included | Low-Chloride Fluid Group | | High-Chloride Fluid Group | | Mean difference |
| --- | --- | --- | --- | --- | --- |
|  | Mean±SD | total | Mean±SD | total |  |
| Weinberg et.al., 2017 | 7.32 ±0.06 | 520 | 7.39±0.05 | 454 | -0.07 (-0.08, -0.06) |
| Pfortmueller et.al., 2018 | 7.37 ^a^ | 30 | 7.29 ^a^ | 30 | / |
| Hadimioglu et.al., 2008 ^b^ | 7.42±0.06 | 30 | 7.44±0.06 | 30 | -0.02 (-0.05, 0.01) |
| Hadimioglu et.al., 2008 ^c^ | 7.42±0.06 | 30 | 7.36 ±0.05 | 30 | 0.06 (0.03, 0.09) |
| O’Malley et.al., 2005 | 7.37± 0.07 | 25 | 7.28± 0.07 | 26 | 0.09 (0.05, 0.13) |
| Khajavi et.al., 2008 | 7.34 ± 0.05 | 26 | 7.29 ± 0.08 | 26 | 0.05 (0.01, 0.09) |
| Takil et.al., 2002 | 7.36± 0.03 | 15 | 7.35± 0.03 | 15 | 0.01 (-0.01, 0.03) |
| Waters et.al., 2001 | 7.40± 0.07 | 33 | 7.35 ±0.09 | 33 | 0.05 (0.01, 0.09) |
| Total/ Summarized concentration | 7.33±0.01 | 709 | 7.38±0.02 | 644 | -0.07 (-0.08, -0.06) |
| I ^2^ | 89.34 | / | 88.89 | / | / |
| Begg | 0.62 | / | 0.33 | / | / |
| Egger | 0.44 | / | 0.20 | / | / |

Abbreviations: SD, standard deviation.

^a^, median.

^b^, plasmalyte group.

^c^, Ringer’s lactate group.

Supplementary Table 8. Summarized serum chloride concentration after surgery in Low- and high Chloride Fluid Groups

| Study included | Low-Chloride Fluid Group | | High-Chloride Fluid Group | | Mean difference |
| --- | --- | --- | --- | --- | --- |
|  | Mean±SD ( mmol/L) | total | Mean±SD ( mmol/L) | total |  |
| O’Malley et.al., 2005 | 106.00±4.00 | 25 | 111.00±4.00 | 26 | -5.00 (-7.20, -2.80) |
| Dey et.al., 2018 | 101.20±4.49 | 22 | 115.91±3.66 | 22 | -14.71 (-17.13, -12.19) |
| O’Malley et.al., 2005 | 106±4.00 | 26 | 111.00±4.00 | 25 | -5.00 (-7.20, -2.80) |
| Takil et.al., 2002 | 109±7.00 | 15 | 115.00 ±5.00 | 15 | -6.00 (-10.35, -1.65) |
| Waters et.al., 2001 | 107±4.00 | 33 | 114.00±6.00 | 33 | -7.00 (-9.46, -4.54) |
| Kim et.al., 2013 | 105.50±4.40 | 30 | 105.50 ±4.10 | 30 | 0.00 (-2.15, 2.15) |
| Hadimioglu et.al., 2008 | 104.00±2.00 | 30 | 125.00±3.00 | 30 | -21.00 (-22.29, -19.71) |
| Total/ Summarized concentration | 105.62±0.88 | 671 | 112.03±1.67 | 605 | -8.99 (-16.69, -1.28) |
| I ^2^ | 90.12 | / | 95.46 | / | / |
| Begg | 0.16 | / | 0.19 | / | / |
| Egger | 0.41 | / | 0.37 | / | / |

Abbreviations: SD, standard deviation.

^a^, range of serum chloride concentration.

Supplementary Table 9. Publication bias of summarized outcomes

| Outcomes | Begg (*P* value) | Egger (P value) |
| --- | --- | --- |
| Summarized mortality in Low- and high Chloride Fluid Groups | 0.43 | 0.76 |
| Summarized renal replacement therapy in Low- and high Chloride Fluid Groups | 0.60 | 0.51 |
| Summarized acute kidney injury in Low- and high Chloride Fluid Groups | 0.71 | 0.60 |
| Summarized use of Allogenic Blood Transfusion in Low- and high Chloride Fluid Groups | 0.98 | 0.93 |
